# Capturing Emergency Contraceptive Pill Use: Critical Reflections on Measurement and Reporting

**DOI:** 10.1111/sifp.70026

**Published:** 2025-07-14

**Authors:** Joe Strong, Ernestina Coast, Jamaica Corker, Michelle Weinberger

## Abstract

Emergency contraceptive pills (ECP) are an essential and unique postcoital method of preventing pregnancy. Trends in supply data show that sales of ECP are increasing at faster rates globally than many other contraceptives. Yet nationally representative survey data suggest that ECP use has remained relatively static overtime, suggesting significant measure and reporting issues. Accurate measurement of ECP use is critical for informing policies and programs that provide people the choice and freedom to exercise their reproductive rights. There is an urgent need for a revision of ECP measurement to better capture the realities of people's contraceptive needs and desires. In this commentary, we outline the key reasons why surveys may be underreporting and misreporting ECP. We focus on issues around current method‐specific measurements, definition issues around “use” and problems with survey questions and prompt phraseology. We illustrate the importance of recognizing other postcoital methods and strategies that people use when trying to prevent a pregnancy, and the implications this has for ECP measurement. As ECP use evolves, we offer recommendations for survey revisions and further research that can ensure that ECP measurement is robust and able to provide accurate reporting in the future.

## INTRODUCTION

Emergency contraception is an essential component of the contraceptive mix that people use to avoid pregnancy. Emergency contraception is unique among contraceptive methods because it provides a mechanism for people to avoid pregnancy postcoitally. Emergency contraceptives can be administered in two different ways. The first is through pills (ECP) that contain either ulipristal acetate or levonorgestrel or through combined oral contraceptive pills. The second is through the insertion of a copper intrauterine device (IUD) (World Health Organization 2021).

The proliferation of ECP and service delivery modes has enhanced accessibility. Globally, people are increasingly using emergency contraception (European Consortium for Emergency Contraception 2023), and the WHO advocates for further increasing the availability of ECP (World Health Organization 2022). As ECP sales and Couple Years of Protection (CYP) are increasing at significantly faster rates than other contraceptives, while survey data report relatively limited and static use (European Consortium for Emergency Contraception 2023), it is necessary to ensure robust and accurate measurement and reporting.

Measurement of ECP use is important for service delivery; it offers insights into the choices, desires, and needs of people seeking to avoid pregnancy. It is also critical for demographic measures such as contraceptive prevalence rate (CPR). If ECP is not measured accurately, its significance for pregnancy prevention is not being captured, limiting funders’ and programs’ abilities to meet people's contraceptive needs. Inaccurate measurement undermines efforts to ensure that people's contraceptive needs and desires are being met to support their full and free informed choices with regard to their sexual and reproductive health, rights (SRHR) and freedoms.

In this commentary, we argue that current methods for measuring emergency contraception use are critically flawed and in urgent need of revision. We provide a brief overview of the unique place of emergency contraception within contraception and family planning. We focus on ECP, which likely accounts for the vast majority of emergency contraception use, given their greater accessibility compared to copper IUDs, the lack of evidence that most IUD use is for EC, and the much higher sales and distribution of ECP (Farid et al. 2024). We outline two key issues: (1) that current survey measurement and reporting may be resulting in significant misreporting of ECP use, most likely underreporting based on the disparities found between sales volumes and survey data; (2) that surveys are failing to capture with specificity what people are doing or using when they respond to questions about “emergency contraception.” We argue that changes in how the use of emergency contraception is being conceptualized by people, and emerging evidence of effective pericoital use of a similar formulation of pill, mean that further refinements are needed in order to advance and future‐proof ECP measurement.

## EMERGENCY CONTRACEPTION: A UNIQUE POSTCOITAL METHOD

Emergency contraception is often treated differently from other contraceptives in SRHR. Since the 2000s, ECP awareness and use have risen significantly across the globe (Weinberger et al. 2024; Awopegba et al. 2021; DKT International FWACA 2024). This has been accompanied by an increase in countries including ECP in their national essential medicines list (USAID 2023) and changes in the regulation and provision of ECP in many countries (Hussain and Kavanaugh 2021). However, unlike other contraceptives, opposition to ECP use, particularly linked to antiabortion opposition, has meant that regulatory reform and efforts to increase access can be complicated and uneven across contexts (Faúndes et al. 2007). The United States offers a contemporary example of the opposition to ECP in the context of broader anti‐abortion and anti‐SRHR politics (Cleland et al. 2022).

The use of the term “emergency” contraception creates a problematic framing that segregates it from other contraceptives considered “regular” (World Health Organization 2021). The language of “emergency contraception” was adopted in health policies and regulations in the 1990s. The definition of an “emergency” refers to three circumstances: when no other contraceptives are used, when contraceptives have failed, or in cases of sexual violence (World Health Organization 1998). Yet, people do not only use emergency contraception exclusively in these situations but also as part of a dual method strategy (Bongomin et al. 2023) or as the sole contraceptive method of choice (Henry et al. 2021; Senders and Horner‐Johnson 2022). People use a variety of language to describe emergency contraception—for example, “morning after pill” (Johnson et al. 2010). “Emergency,” therefore, contrasts people's own language and reasons for use, and people may not distinctly separate ECP from “regular” contraceptives.

ECPs are also different compared to other modern methods in their main procurement channel. In contrast to other contraceptives, the vast majority of ECP provision in countries that receive family planning assistance is through commercial and socially marketed products (Weinberger et al. 2024). In low‐income countries, the public sector accounts for more than 80 percent of the commodity costs for the two most widely used methods—injectables and implants.  By contrast, more than 90 percent of ECP costs are incurred in the private sector (Weinberger et al. 2024). Donors procure very few ECPs; of all contraceptive supplies tracked by RHViz for 2023, less than 1 percent (USD 1.5 million out of a total of 230.7 million) was spent on EC (Weinberger et al. 2024). UNFPA Supplies spent only USD 708,000 of 112 million on ECP, UNFPA co‐financing USD 438,000 of 28 million, and USAID USD 273,000 of 55 million (Reproductive Health Supplies Coalition). This gives less visibility to ECP procurement and sales compared to other methods and may also affect the prioritization of efforts to increase this visibility.

## CURRENT MEASURES UNDERREPORTING EMERGENCY CONTRACEPTION USE

Recent supply‐side data show substantial increases in ECP procurement and distribution. The Contraceptive Social Marketing Statistics, which tracks sales of contraceptives across more than 70 programs, show a rapid increase from 8.9 million cycles sold in 2010 to 39.1 million in 2023, an increase of 340 percent (Reproductive Health Supplies Coalition 2024). These estimates do not capture non‐socially marketed products, which make up a sizable share of the ECP market in many countries (Weinberger et al. 2024).

Despite this substantial growth in ECP sales and distribution, the estimated use of ECP in recent years has remained relatively stable across nationally representative surveys (ICF 2024). While some of this increase in supply may be due to greater use by existing ECP users or lags between distribution and use, it is highly unlikely that these alone account for the discrepancy between substantial increases in ECP distribution and relatively little change in reported use. A recent analysis of the contraceptive market across 132 low‐ and middle‐income countries (LMICs) estimated that using demographic measures—contraceptive use and method mix—captures only 22 percent of the emergency contraceptive market in Sub‐Saharan Africa and 6 percent among other LMICs, compared to estimating the market value through supply‐side approaches (Weinberger et al. 2024). This analysis, coupled with the disparity between supply and use reports, strongly suggests critical measurement issues and underreporting of ECP use in nationally representative surveys.

There are three key reasons why ECP may be underreported in nationally representative surveys: (i) how surveys report method‐specific prevalence rates; (ii) restrictive definitions of “contraceptive use”; (iii) phrasing of ECP in survey questions and conceptual challenges around the term “emergency.” For method‐specific prevalence rates—such as “current use”—most surveys report only the “most effective” method when a respondent reports the use of multiple methods (Farid et al. 2024). This potentially biases downward estimates of ECP use when used in tandem with other more effective methods. Standard CPR and method mix estimates, therefore, do not capture ECP used as part of a broader strategy of pregnancy prevention.

Restrictive definitions of contraceptive use, namely measuring current use as within the past month, may also lead to ECP underreporting by surveys. Research on definitional changes to the measurement of ECP by Larson et al. (2020) highlights that more inclusive definitions of ECP, which incorporate use in the last 12 months as well as concurrent use with more effective methods, increase overall estimates of use. Yet even this expansive time frame is likely still not capturing the full picture of ECP use. People may not consider ECP a method of family planning in the same way as other contraceptives, and therefore not report that their ECP use was a “current” mechanism to “delay or avoid getting pregnant” (ICF 2023). This may mean that respondents underreport use in questions on ECP. Additionally, recent author experiences conducting research on ECP found that survey questions that ask about methods used at the “(last) time you had sexual intercourse” (ICF 2023, 52) may not resonate if a respondent took ECP days after they had sex, which could further contribute to underreporting or ECP use.

A broader and more inclusive range of questions and data used to define “use” may help more accurately measure ECP use. For example, the addition of Q306 to Phase VIII of the DHS, which probes specifically around coitus‐dependent methods including emergency contraception, allows further data points to assess the scale of underreporting in current measurements (ICF 2020). This additional question is specifically to account for people not initially framing these methods, including ECP, as a form of family planning or contraceptive, as they might with other methods (ICF 2020). A comprehensive analysis of the differences in reporting is needed to examine the utility of this probe in more accurately measuring ECP use.

Phrasing around ECP in survey questions and interview prompts may also lead to misreporting. Many surveys ask questions on “emergency contraception”—which is a broad umbrella term—when they are actually seeking information on ECP. For example, the DHS interview training manual explicitly instructs interviewers to not count someone who reports using an emergency intrauterine device (ICF 2020), despite the English‐language questionnaire referencing “emergency contraception” (ICF 2023, 10). For many people, the phrase “emergency contraception” may not be ubiquitous, and terminology can vary across languages, as well as contexts. In French‐language surveys, including PMA2020 (multiple countries) and FECOND (France), “la pilule du lendemain” [the morning‐after pill] has been used in tandem with “contraception d'urgence” [emergency contraception] (Bajos and Moreau 2022; Institut Supérieur des Sciences de la Population (ISSP), Université Joseph Ki‐Zerbo, and Bill & Melinda Gates Institute for Population and Reproductive Health at the Johns Hopkins Bloomberg School of Public Health 2019). “Morning‐after pill” appears in DHS interviewer training manuals, but not in the English‐language questionnaires (ICF 2020, 78).

Phraseology can become more complicated as a result of survey prompts. In the DHS, the question prompt refers to ECP as follows: “an emergency measure, within three days after they have unprotected sexual intercourse, women can take special pills to prevent pregnancy” (ICF 2023, 10). There are critical issues with this prompt. There is an inherent ambiguity as to what is understood by “special pills.” Without offering contextually relevant brand names, it is not possible to know whether people's responses reflect ECP or other non‐ECP pills. In some contexts, a wide range of pharmaceutical pills are taken postcoitally as part of pregnancy prevention strategies (Ebuehi, Ekanem, and Ebuehi 2006), some of which may not actually be effective in preventing pregnancy. ECP use in the context of “unprotected sex” may misalign with a person's own reasons for use (Teixeira et al. 2012; Marston et al. 2017; Strong 2024). People may be using contraception and still use ECP, or they may consider that their sex was protected because they had planned for ECP use. Moreover, the questions and prompts raise conceptual challenges around the term “emergency” that may challenge the accurate measurement of ECP use. Evidence highlights that people use ECP outside of “emergency” circumstances, including planned use (Teixeira et al. 2012).

Developing questions and prompts that ask specifically what emergency contraception is being used may be a useful way of ensuring that the data captured reflects actual methods of EC. If we want to know about ECP use, then surveys—questions, prompts, interviewers—need to unambiguously identify ECP. The US‐based National Survey of Family Growth, for example, uses prompts that include specific brands and names of emergency contraceptive pills as a way of supporting a person's understanding and recall (National Center for Health Statistics 2021). If a respondent has never heard of ECP, many current survey prompts may not hold resonance or relevance and therefore not work effectively at capturing whether they are, in fact, using ECP. Reliance on the language of “emergency contraception” or probes that tie ECP to being an “emergency measure” may not align with people's own understandings and vocabularies (Strong 2024), thereby reducing potential reporting. Ultimately, if surveys intend to collect data only on emergency contraception pills, they should be explicit in doing so.

**Case Study: Measuring Emergency Contraceptive Pill Use in Ghana**

Ghana offers a useful—purposefully selected—example of the current issues of understanding ECP use from diverse evidence sources. Supply‐side data from DKT's social marketing of ECP in Ghana, where 98 percent of ECP users report accessing ECP via private medical sector non‐NGO sources (Ghana Statistical Service and The D. H. S. Program 2024), show a 10‐fold increase in the volume of ECP sales between 2014 and 2022 (0.45 million and 4.7 million, respectively) (DKT International 2023).

Nationally representative DHS survey data, on the other hand, do not seem to indicate levels of use expected for over 4 million ECP sales annually—or 200,000 CYP—with only 2.8 percent of all women reporting ECP as their current (and most effective) method (Ghana Statistical Service and The D. H. S. Program 2024); with higher use reported among sexually active unmarried women (11.5 percent) as compared to married women (1.1 percent).  DHS comparisons with previous surveys are hindered as the 2014 survey does not report on ECP and the 2003 and 2008 surveys only report ever‐use of ECP. PMA2020 data from 2013 to 2017 show only modest increases in ECP's role in the modern method mix between Round 1 (2013) and Round 6 (2017); from 2.7 percent to 4.4 percent of the married women's modern method mix, and 15.6 percent to 18.9 percent of the unmarried women's modern method mix (Performance Monitoring and Accountability 2020 (PMA2020) Project and Kwame Nkrumah University of Science and Technology (KNUST) 2013, 2017). Nationally, the 2022 DHS found that men and women had similar levels of knowledge of ECP—75.5 percent of women and 70.6 percent of men reporting having heard of ECP (General Directorate of Statistics [Ghana], Ministry of Finance [Ghana], and ICF 2022).

Two probing questions about ECP were introduced in Round 8 of the DHS, including the 2022 Ghana DHS, one probing for the use of coitally dependent methods (including ECP) and one asking about any use of emergency contraception in the last 12 months (Ghana Statistical Service and The D. H. S. Program 2024). We find that the probe on coitally dependent methods may have resulted in little change in reported ECP use and suggest follow‐up analysis to examine this further. Asking about use in the past 12 months, however, solicits some additional reported ECP use, increasing from 11.5 percent current use to 26.9 percent in the past 12 months among unmarried sexually active women. Yet even this probing question is likely not capturing all ECP use as responses illustrate conceptualization challenges: comparing responses across ECP questions shows that 26 women who reported never using contraception report having used ECP in the last 12 months (out of a total of 1226 women reporting use of ECP in the last 12 months), and 93 women who reported using ECP at last sexual intercourse report they are “not currently” using any method of contraception (out of a total of 415 women reporting use of ECP at last sexual intercourse) (General Directorate of Statistics [Ghana], Ministry of Finance [Ghana], and ICF 2022). 

A range of observational studies with diverse populations across Ghana suggest higher levels of ever‐use of ECP than reported in nationally representative surveys (Addo and Tagoe‐Darko 2009; Opoku and Kwaununu 2011; Amalba et al. 2014; Rokicki and Merten 2018; Mohammed, Abdulai, and Iddrisu 2019; Ngmenbelle et al. 2024). In a study of final‐year nursing and midwifery students, a quarter (25.65 percent) reported ever‐use of ECP and an additional 16.8 percent were unsure or could not remember if they had ever used ECP (Mohammed, Abdulai, and Iddrisu 2019). In a study of female students at a tertiary university, 48 percent of respondents reported the use of ECPs (Ngmenbelle et al. 2024). Additionally, a study using Ghana Health Service District Health Information Management System data showed substantial increases in the number of ECP users during COVID‐19, adding to an upward trend in ECP use pre‐COVID‐19 (Fuseini et al. 2022) that does not seem to be reflected in the GDHS 2022. 

Evidence highlights the wide range of non‐ECP pharmaceuticals and other substances and methods that are reported in response to questions about ECP (Opoku and Kwaununu 2011; Rokicki and Merten 2018). For example, unmarried sexually active women aged 18–24 interviewed in Accra reported urinating or washing after sex as a method of preventing pregnancy postcoitally (Rokicki and Merten 2018). Further, the use of the term “emergency” can be problematic for measurement. For example, evidence from a survey among men in Accra found that respondents were much less familiar with the term “emergency contraception” than with descriptions of ECP use or brand names. Men associated ECP with pleasure, condomless sex, and intimacy (Strong 2024). The same study found that men might buy pills to either facilitate their partner's choice of contraception or to exert pressure on a partner (Strong 2024). This raises the possibility that women may unknowingly receive ECP from partners, which could affect accurate reporting

## CAPTURING THE BROADER RANGE OF POSTCOITAL METHODS OF PREGNANCY AVOIDANCE

While it is important to understand people's use of purposely formulated emergency contraception, as these represent the safest and most effective methods of postcoital contraceptives available to them, it is equally important to capture the use of other postcoital methods of pregnancy avoidance, regardless of their efficacy. Data on any and all use of postcoital strategies are indicative of people's desires to avoid pregnancy as well as a demand for these methods. Moreover, it provides necessary evidence on the potentially less effective (or ineffective) methods that people may be using, including those that are harmful to their health, providing crucial information needed to inform comprehensive counseling, service delivery interventions, and healthcare provision.

There is enormous variation in what people use as postcoital contraception. Alongside the use of purposely formulated ECP, evidence illustrates many other pharmaceutical and nonpharmaceutical methods used (Teixeira et al. 2012). This includes other pharmaceutical pills such as antibiotics (Lathrop et al. 2013) or paracetamol (Teixeira et al. 2012). Congolese women refugees in Uganda, for example, used antimalarials and analgesics to try to prevent pregnancy after sex (Nara, Banura, and Foster 2020). This further complicates existing ECP measures; people may respond to existing questions (and prompts) that they are taking a pill that they consider to be a postcoital contraceptive, up to 3–5 days after sex. Such responses may erroneously record the use of these other non‐ECP pills as emergency contraception. We recommend research that will allow for estimations of the extent to which these types of other pharmaceutical and nonpharmaceutical methods are being used.

People use a variety of nonpharmaceutical methods, such as drinking large volumes of salt/cold water after sex (Lathrop et al. 2013), enemas (Baiden, Awini, and Clerk 2002), douching (Addo and Tagoe‐Darko 2009), drinking certain liquids (Teixeira et al. 2012), among others, to prevent pregnancy after sex. It is not clear in the current data collection whether this would be reported and recorded as “emergency contraception,” “other” method, or not at all. The ability to capture nuanced data that can parse out the full range of postcoital methods people use gives a more granular and necessary assessment of where awareness‐raising efforts and emergency contraception service provision may be wanted and needed. This disaggregation could be achieved through further research and the development of a targeted set of questions addressing postcoital methods that are specific to these various categories: purposely formulated ECP, other (noncontraceptive) pharmaceuticals, and nonpharmaceuticals.

## EVOLVING OUR MEASUREMENT OF EMERGENCY CONTRACEPTION

We argue the urgent need for a more critical review of research methods to measure emergency contraceptive use. Emergency contraceptive pills are a growing and significant component of people's strategies to prevent pregnancy. Current measurement does not capture the extent of ECP use nor the complexities of other postcoital contraceptive strategies, significantly limiting the capacity for programs and services to meet people's contraceptive needs and desires. Without this, people continue to face barriers to exercising their rights and making reproductive decisions freely. Revising ECP measures is not only needed to ensure that people's rights are met but also to provide the necessary data for contraceptive suppliers and funders to ensure funding and provision reflect choices and demands.

Further research is needed to evidence where misreporting is most often occurring and to design and test questions that can elicit greater precision. Uncovering critical areas of misreporting is necessary for making decisions about the most effective way of improving EC data collection. We acknowledge that our call for improving EC measurement comes at a time when the field is facing uncertainty about the future availability of data on contraceptive use, given the defunding of DHS (Ghany et al. 2025). However, other survey mechanisms and research projects will benefit from investments in improving EC measurement. Studies such as the AFIDEP‐led “Re‐examining Traditional Method Use (TEAM‐UP)” project offer rich empirical data on postcoital practices that can be used in the development of new measures of EC that may better capture people's practices. Mapping the ways in which EC is asked about in surveys from all global contexts allows a useful roadmap for creating new questions.

Emergency contraception is important for people across the globe and is a critical part of people's contraceptive strategies. In order to meet people where they are, and support them to fulfill their choices, desires, and needs, it is necessary to understand their current practices and behaviors. We recommend cognitive research into how ECP and postcoital methods questions are understood and answered across languages, contexts, and populations and developing new and unambiguous nomenclature around ECP, postcoital, and peri‐coital methods. This includes critical reflections on the utility and relevance of the word “emergency” and potential methods to capture “planned” versus “emergency” use.

Despite recent additions of questions on ECP in key nationally representative surveys, further prioritization of revisions to ECP questions is needed. This includes redefining and reconceptualizing reporting of “use” for ECP (and other coitally dependent methods), with clear and comprehensible definitions and questions, including contextually and linguistically relevant prompts, to ensure that measures are able to disaggregate increasingly overlapping methods. The renewed development of peri‐coital contraception (Bell et al. 2024)—pills that can be taken shortly before or after sex—blur definitions of what constitutes emergency contraception or other postcoital methods of pregnancy prevention. This means questions need to revise how they address temporal issues, to help understand which contraceptive is being used. New measures would ideally be compatible with existing measures, notably CPR so ECP's role in overall contraceptive use can be highlighted, while also introducing new measures that more accurately capture emergency contraception to better understand both current use patterns and needs.

We urge the family planning/contraception field—researchers, program designers, policymakers— to prioritize revising and reconceptualizing how we define and measure emergency contraception and other postcoital methods beyond surveys. Future research, funding, and programs must improve targeted measurement of the range of postcoital methods people use to complement current understandings of emergency contraceptive use and to better inform comprehensive and rights‐based family planning and reproductive health programs.

## ETHICS APPROVAL STATEMENT

No ethics approval is required.

## PATIENT CONSENT STATEMENT

No patient consent is required.

## PERMISSION TO REPRODUCE MATERIAL FROM OTHER SOURCES

No permission was required.

## CONFLICT OF INTEREST DISCLOSURE

The authors report no conflict of interest.

## Data Availability

All data quoted in this article are from published reports. No original data are used.
